# Is Hartmann’s Pouch an Option in the Management of Acute Severe Ulcerative Colitis?

**DOI:** 10.3390/jcm11133857

**Published:** 2022-07-03

**Authors:** Myriam Renaud, Ahmet Ayav, Bénédicte Caron, Laurent Peyrin-Biroulet, Adeline Germain

**Affiliations:** 1 Department of Hepatobiliary, Colorectal and Digestive Surgery, Nancy University Hospital, University of Lorraine, 54500 Vandoeuvre-lès-Nancy, France; 2 Department of Gastroenterology, Nancy University Hospital, University of Lorraine, 54500 Vandoeuvre-lès-Nancy, France; 3 Inserm NGERE U1256, Nancy University Hospital, University of Lorraine, 54500 Vandoeuvre-lès-Nancy, France

**Keywords:** colectomy, surgical stomas, morbidity, digestive-system surgical procedures, ulcerative colitis

## Abstract

Background: The surgical management of remnant rectosigmoid after subtotal colectomy with end ileostomy for acute severe ulcerative colitis remains controversial with respect to the need to perform sigmoidostomy or Hartmann’s pouch. The aim of this retrospective study was to investigate whether Hartmann’s pouch may be a safe option. Methods: Thirty-eight Hartmann’s pouches were performed between January 2003 and December 2015. We looked at morbidity (with a focus on the occurrence of pelvic sepsis and leakage of the rectal stump) and the rate of restored intestinal continuity. Results: Nineteen patients had surgical complications. Seven had an intra-abdominal collection, only one of which was in the pelvis, and the patient had to be reoperated. Only one patient had a reopening of the rectal stump, which was revealed by rectal bleeding. Twenty-six patients (68.4%) underwent further proctectomy with ileal J-pouch anal anastomosis with no difficulty in localizing or mobilizing the rectal stump and no major surgical complications. Conclusions: Hartmann’s pouch may be considered in patients with acute severe ulcerative colitis, with low rates of morbidity and pelvic sepsis. The restoration of intestinal continuity is possible after this procedure without any special difficulty.

## 1. Introduction

Approximately 15% of patients with ulcerative colitis (UC) develop an acute attack of severe colitis according to Truelove and Witts criteria [1]. Intravenous steroids remain the first medication for acute severe ulcerative colitis (ASUC) management [2,3]. In case of steroids failure, second-line therapy with either ciclosporin or infliximab may be initiated [4]. A multidisciplinary approach between gastroenterologists and surgeons is essential to determine the time of surgery [3,5,6,7]. Surgery is necessary when there is no improvement despite optimal medical therapy or when a complication arises (colonic perforation, massive hemorrhage, toxic megacolon) [8,9,10,11].

A procedure in three stages, with subtotal colectomy with end ileostomy as the first stage, followed by coloproctectomy with ileal J-pouch anal anastomosis (IPAA) with covering loop as the second stage, and the closure of covering loop ileostomy as the third stage, is the gold standard for the management of ASUC [3]. However, there is no recommendation for the management of the retained rectal segment after subtotal colectomy [3,12]. There are several possibilities: an intraperitoneal closure of the rectal stump such as Hartmann’s pouch [13,14,15,16,17], the subcutaneous placement of the retained colorectal segment [18,19,20,21], or the realization of sigmoidostomy [22,23]. Some authors have argued against the creation of an intra-abdominal Hartmann’s pouch due to the risk of pelvic sepsis secondary to the leakage of the rectal stump, difficulties in localizing the rectal remnant segment, and potential dissection difficulties during proctocolectomy and IPAA procedure [19].

In our department, Hartmann’s pouch is the procedure of choice and has been used for many years. The aim of this retrospective single-center study was to evaluate the rate of postoperative complications, with a focus on intra-abdominal septic complications, and the rate of restorative proctocolectomy after Hartmann’s procedure for ASUC.

## 2. Material and Methods

### 2.1. Patient Selection

We performed a retrospective study including all consecutive adult patients who underwent subtotal colectomy with Brooke ileostomy and the intraperitoneal closure of the rectosigmoid in an emergency setting for ASUC between January 2003 and December 2015 in the Department of General Surgery at Nancy University Hospital. All patients had ASUC according to Truelove and Witts criteria that was refractory to optimal medical treatment or complicated by hemorrhage, colonic perforation, or toxic megacolon.

Patients for whom the diagnosis of UC was not confirmed after the pathological analysis of the surgical specimen and who had surgery for an indication other than ASUC were excluded from the study.

### 2.2. Data Collection

Data were extracted from each patient’s medical and anesthesia charts: age, gender, BMI (Body Mass Index), weight loss, smoking, ASA (American Society of Anesthesiologists) score, date of UC diagnosis, Montreal classification [24], medications, disease duration at time of surgery, preoperative biological parameters (hemoglobin, C-reactive protein, and albumin levels), perioperative antibiotics treatment, surgical history, characteristics of the surgical procedure, operative time, intraoperative and postoperative complications, length of hospital stay, and duration between each surgical step.

BMI and albumin level were used to define each patient’s corpulence and malnourishment state [25,26]. The operative time included all phases from incision to skin closure. The complications were classified according to the Clavien–Dindo classification [27].

Categorical variables were described as percentage, while continuous variables were reported as mean ± standard deviation (SD) or median [interquartile range (IQR)], depending on their distribution.

### 2.3. Technical Considerations

The operation is usually undertaken in 3 steps, especially in patients receiving corticosteroids or immunomodulators, with poor general health status, and/or with undernutrition at the time of surgery.

First stage: Subtotal colectomy with Brooke ileostomy [3,8,28,29].

Surgical treatment consists of a subtotal colectomy procedure that is carried out along the colon, associated with an intraperitoneal Hartmann’s closure of the rectum remnant at the sacral promontory level with a stapler and a standard end ileostomy in the right iliac fossa.

Second stage: Additional coloproctectomy and restorative IPAA with covering loop ileostomy [3,8,28].

The surgical approach for the first and second steps is laparoscopy or laparotomy.

Third stage: Closure of covering loop ileostomy by local approach at 2–3 months, usually after a systematic control of the pouch by radiological opacification through ileostomy in order to rule out anastomotic leakage or anastomotic stenosis.

## 3. Results

Thirty-eight patients underwent subtotal colectomy with terminal ileostomy and a closure of the retained rectosigmoid pouch for ASUC between January 2003 and December 2015 in our department.

### 3.1. Baseline Characteristics

There were 19 males (50%). The mean age at diagnosis was 33 years (15), and the mean age at surgery was 38 years (15). The median follow-up was 13.0 months (19), while the mean follow-up was 20.5 months (19.4). At the time of the first surgery, 10.5% of the patients were smokers, and 26.3% of the patients were former smokers (Table 1).

The mean albumin level was 26.7 g/L (6.5). Undernutrition (albumin level < 25 g/L) was found in 28.9% (11/38) of patients. Only 15.8% (6/38) of patients had a BMI < 18.5 kg/m^2^. Weight was normal in 60.5% of the patients, and 25.9% of patients were overweight.

The ASA scores were 2 and 3 in 71.1% and 28.9% of patients, respectively.

All patients had extensive colitis (E3 according to the Montreal classification). They all had ASUC according to the criteria of Truelove and Witts.

The median disease duration was 48.9 months (50.9) at time of surgery. UC had been diagnosed less than one year prior in 23.7% of the cases and more than five years prior in 28.9% of the cases. A surgical procedure was required for the first flare-up of UC in six patients (15.8%).

Eight patients (21%) had had at least one previous major abdominal surgery. One patient underwent sigmoidectomy for diverticulitis. Other procedures were: three Caesarian procedures, three abdominal wall surgeries, one cholecystectomy procedure by laparoscopy, and one annexectomy procedure by an open approach.

Indications for surgery were lack of response to optimal medical treatment for 33 patients (86.8%) and disease complications for the five remaining ones (two cases of uncontrolled massive hemorrhage (5.3%), one case of colonic perforation (2.6%), and two cases of toxic megacolon (5.3%)).

The diagnosis of UC was confirmed by histological analysis for all included patients.

### 3.2. Medical Treatment of ASUC

All patients received intravenous corticosteroid therapy for ASUC. Aminosalicylates were used in 63.1% (past medications, 42.1%; concomitant medications, 21.0%) and thiopurines (azathioprine) in 73.7% of our patients (past medications, 52.6%; concomitant medications, 21.0%).

Second-line treatment was ciclosporin, which was used in 36.8% of cases (14 patients).

Twenty patients (52.6%) were on anti-TNF treatment (infliximab or adalimumab) at the time of surgery. Three patients (7.9%) had previous infliximab exposure. Four patients received both anti-TNF agents.

Methotrexate was used in 5.3% (2/38) of cases (one past medication and one concomitant medication).

Twenty patients (52.6%) received at least one pouch of packed red blood cells (from 1 to 12 units).

Preoperative antibiotic treatment was administrated to 63.1% of the patients.

### 3.3. Surgical Management of ASUC

A laparoscopic approach was used in 30 patients (78.9%), with no need for conversion, and an open approach was adopted in 8 patients (21.1%). The median total operative time was 140 min (106), 125 min in case of an open approach and 195 min in case of a laparoscopic approach.

### 3.4. Postoperative Course

#### 3.4.1. Early Complications (30 Days after Surgery)

There were no deaths in our study population. Nineteen patients (50%) experienced complications in the 30 postoperative days.

Eleven patients (28.9%) had a complication that was rated superior to grade II according to the Clavien–Dindo classification [27]. A surgical complication occurred in 16 patients (42.1%). Seven patients (18.5%) had an intra-abdominal collection, and only one was located in the pelvis. Five patients underwent percutaneous drainage, and one patient underwent surgical drainage of a pelvic collection. Bacteria culture was only positive in two cases.

During the same period, we performed five subtotal colectomy procedures with sigmoidostomy, and two of the five patients developed intra-abdominal collections (40%).

The characteristics of intra-abdominal collections are detailed in Table 2.

Only one patient (2.6%) had a reopening of the rectal stump, which manifested by rectal bleeding and required a redo surgery. Three patients had postoperative ileus only requiring medical treatment. One patient had an enterocutaneous fistula around the ileostomy location, requiring a redo surgery. Eight patients (21.0%) had a medical complication.

A reoperation was performed in four patients (10.5%) in the 30 postoperative days: one patient underwent a surgical drainage of a pelvic collection on day 4; one patient underwent a surgical drainage of a reopening of the rectal stump on day 7; one patient underwent laparoscopy for small-bowel obstruction on day 9; and one patient had ileostomy repair on day 24.

The median length of hospital stay was 14 days (10). It was 13 days for patients who underwent subtotal colectomy with ileostomy and Hartmann’s pouch by laparoscopy versus 17.5 days for those operated on with an open approach.

#### 3.4.2. Late Complications (>30 Days)

A seventeen-year-old woman experienced two complications. She presented, on day 32, with peritonitis related to a volvulus of the small bowel requiring reoperation with an intestinal resection of 75 cm. This patient experienced a small-bowel occlusion in the fourth month, and she was reoperated.

### 3.5. Restoration of Intestinal Continuity

**Figure 1:**
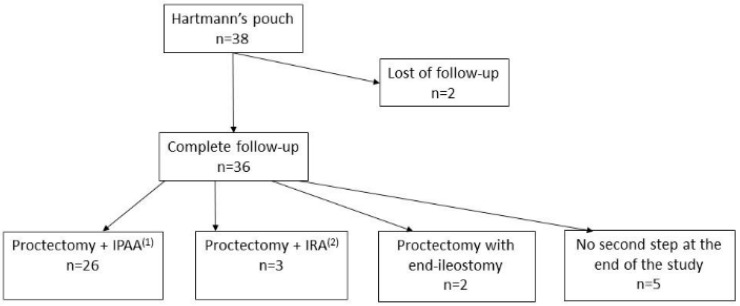
Flowchart. ^(1)^ Ileal J-pouch anal anastomosis. ^(2)^ Ileorectal anastomosis.

Two patients were lost at follow-up. Out of 36 remaining patients, intestinal continuity was restored in 29 patients (76.3%), after a median duration of 4.1 months (2.3). Twenty-six patients (68.4%) underwent proctectomy with IPAA, and three patients (7.9%) underwent ileorectal anastomosis without stoma. Two patients (5.3%) underwent proctectomy with definitive end ileostomy, where one patient was a 70-year-old man who preferred no further surgery beyond ileostomy, while the other patient had a BMI > 30 kg/m^2^, which made it technically impossible to perform IPAA. Nine patients (23.7%) did not undergo a second surgical step; general anesthesia was contra-indicated in one patient, while eight refused further surgery (Figure 1).

Proctectomy with IPAA was performed by laparoscopy in 17 patients (65.4%) and by laparotomy in 9 patients (34.6%). Four patients had previously had laparotomy for subtotal colectomy, and five patients underwent a conversion to the open approach because of adhesions.

Ileorectal anastomosis was performed by laparoscopy in one patient (33.3%) and by laparotomy in two patients (66.6%).

No mortality and no surgical morbidities superior to grade II according to the Clavien–Dindo classification were observed after the second surgical step.

The median length of hospital stay after restorative surgery was 10 days (3).

A total of 25 out of 26 patients with IPAA and covering loop ileostomy underwent the third surgical step. The last patient was lost at follow-up. The median duration between the last two steps was 78 days (22). The median length of hospital stay after closing loop ileostomy was 5.5 days (2). There were no major surgical complications.

## 4. Discussion

Approximately 15% of patients with UC have ASUC. In case of the failure of medical treatment, including ciclosporin or infliximab, surgery should be considered [3]. It is recommended, nowadays, to perform a stage procedure with colectomy first and then end ileostomy. However, the best surgical management of the rectal remnant for these patients is unknown. Several types of surgical procedures have been proposed in the literature, such as an intraperitoneal closure of the rectal stump such as Hartmann’s pouch [13,14,15,16,17], the subcutaneous placement of the retained colorectal segment [18,19,20,21], or the performance of sigmoidostomy [22,23].

The choice regarding the type of surgical procedure essentially depends on the experience of the surgeon and/or of the inflammatory bowel disease (IBD) center. In our department of Digestive Surgery at Nancy University Hospital, we usually perform Hartmann’s procedure as previously described with the intraperitoneal closure of the rectum remnant at the sacral promontory level. During the same period, only five sigmoidostomy procedures were performed. This approach is justified because sigmoidostomy may not eliminate the risk of sepsis [23]. Furthermore, the patients are exposed to a double risk of stoma complications such as invagination, stoma prolapse, or, in the long term, postoperative hernia. Local morbidities (wound infection or abscess) are more important than in the case of Hartmann’s surgery [14,15,16]. The length of pathological colon left is also greater in the case of sigmoidostomy. The control of symptoms (such as persistent mucus discharge or bleeding from the retained diseased bowel) is more difficult than when only a rectal stump is present. The second stoma may also deteriorate a patient’s self-image. Furthermore, there may be some problems of stoma appliance when sigmoidostomy is exteriorized in the same opening as ileostomy.

It has been argued against Hartmann’s pouch that this procedure would be responsible for a higher pelvic sepsis rate, in particular because of the risk of a reopening of the rectosigmoid stump [19]. In 1995, Karch et al., and other authors thereafter, showed that Hartmann’s pouch could be performed in the case of inflammatory bowel disease because the rectal stump reopening rate and pelvic sepsis rate were acceptable [14,15,16]. In our series, the rate of pelvic infectious complications was 2.6%. This rate is lower than the rates reported in the majority of studies published over the last decade (from 3.2% to 6.9%) [16,17,20,21,30]. The stump reopening rate was also 2.6% in our population. This rate is similar to those previously reported (from 0% to 7.4%) [16,17,20,21,30].

Seven patients (18.4%) developed intra-abdominal collections. None was due to a reopening of the rectal stump. During the same period, we performed five subtotal colectomy procedures with sigmoidostomy, and two of the five patients developed intra-abdominal collections (40%). In our series, pelvic sepsis is likely explained by a secondary infection of a lymphatic collection and not by the leakage of the rectal stump. The extended dissection and the inflammatory aspect of the colon in this context could explain the postoperative lymphatic collections, even if no lymph node dissection was performed.

Mortality rates from 0% to 5,2% have been reported in the postoperative period after the surgical management of ASUC [14,15,16,20,21,31,32]. There was no mortality rate in our study after performing Hartmann’s procedure. However, our global morbidity rate was relatively high since 51.9% patients had a complication in the postoperative course. Patel et al. also described a high morbidity rate [33]. This result could be explained by the fact that patients were in a poor physical condition at the time of surgery. While the mean BMI was normal, 28.9% were undernourished. Furthermore, in our study, all patients received intravenous corticosteroids, and for most of them, second-line treatment. There were no defined protocols for stopping corticosteroids after surgery; this was left to the discretion of the physician. Twenty patients (52.6%) were exposed to anti-TNF-α. This rate is higher than that in the literature [14,16,17,20,21,23,30,31]. As these patients were operated on in the biologics era (2003–2015), this could explain why a high rate of postoperative complications was observed [34].

The surgical procedure recommended for the second operating step is coloproctectomy with the restoration of intestinal continuity by ileal J-pouch anal anastomosis [3]. This procedure gives satisfaction and an acceptable quality of life to these patients [17].

This surgery was performed in 26 (68.4%) patients without any difficulty in localizing and mobilizing the rectal stump, as already noted before [15,16] and contrary to the criticism that some surgical teams have made on this subject [19,23]. It was performed by laparoscopy in 17 (65.4%) patients. Laparotomy was the surgical approach when patients had already had laparotomy or when laparoscopy was impossible because of important abdominal adhesions. For Holubar et al. [35], laparoscopy seems to be a safe and feasible surgical approach to perform coloproctectomy with IPAA. It was confirmed by our results.

This study has some limitations. It is a retrospective study of relatively small sample size. In addition, no control group was available, as only five patients underwent sigmoidostomy during the same period in our center.

The strengths of this study are the fact that it was conducted in the biologics era and that the laparoscopic approach was adopted in 30 (78.9%) patients.

In conclusion, our study suggests that an intra-abdominal Hartmann’s pouch in patients with ASUC, in particular by laparoscopic approach, is not associated with a high rate of pelvic sepsis or difficulty in mobilizing the rectal stump and involves no mortality. This surgical approach is feasible and safe with low morbidity–mortality rates. The restoration of intestinal continuity with IPAA is possible after this procedure.

## Figures and Tables

**Table 1:** Patient characteristics.

Patient Characteristics	n = 38 (%)
Sex (male/female)	19/19 (50/50)
Mean age at surgery, years (SD) Mean follow-up, months (SD)	38 (15) 20.5 (19.4)
Smoking	
Smoker	4 (10.5)
Former smoker	10 (26.3)
Non-smoker	24 (63.2)
Mean BMI (kg/m^2^)	22.2
Mean albuminemia (g/L)	26.7
ASA score	
2	27 (71.1)
3	11 (28.9)

**Table 2:** Characteristics of intra-abdominal collections.

Location	Finding Date	Collection Aspect	Drainage	Bacteria Culture
Subhepatic	Day 8	Sterile lemon-colored liquid	None	N/A
Left side	Day 25	Infected hematoma	Percutaneous drainage	Positive
Left side	Day 5	Lymphatic collection	Percutaneous drainage	Negative
Left side and omental sac	Day 29	Light liquid	Percutaneous drainage	Negative
Pelvis	Day 4	Secondary infection of lymphatic collection	Surgical drainage	Positive
Left side	Day 8	Lymphatic collection	Percutaneous drainage	Negative
Perihepatic	Day 5	Lymphatic collection	Percutaneous drainage	Negative

## Data Availability

The data underlying this article are available in the article.

## Abbreviations

ASA (American Society of Anesthesiologists), ASUC (acute severe ulcerative colitis), BMI (Body Mass Index), IBD (inflammatory bowel disease), IPAA (ileal J-pouch anal anastomosis), UC (ulcerative colitis).
